# Supplementary material for: Withasomniferol C, a new potential SARS-CoV-2 main protease inhibitor from the Withania somnifera plant proposed by in silico approaches
Source: PeerJ. 2022 Jun 2;10:e13374. doi: 10.7717/peerj.13374 (PMC9167582; doi:10.7717/peerj.13374)
Supplement: Supplemental Information 6 [file peerj-10-13374-s006.docx]

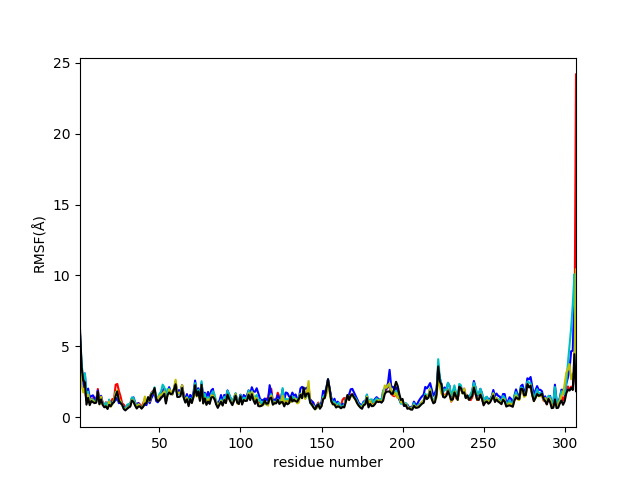


Supplementary figure 1. RMSF plot of 3CL^pro^ complexes with ligands WS1 (red), WS4 (blue), WS7_v1 (cyan), WS7_v2 (olive), WS11 (black).


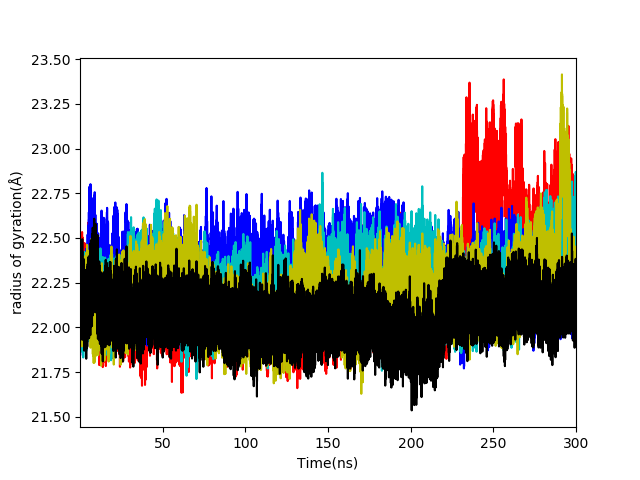


Supplementary figure 2. The radius of gyration plot of 3CL^pro^ complexes with ligands WS1 (red), WS4 (blue), WS7_v1 (cyan), WS7_v2 (olive), WS11 (black).


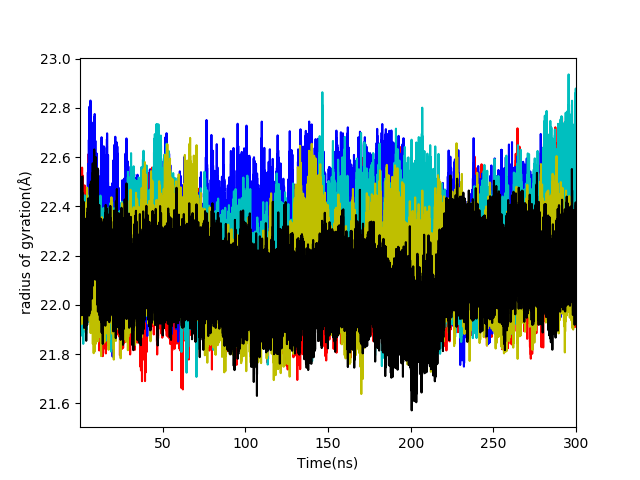


Supplementary figure 3. The radius of gyration plot of 3CL^pro^ receptor of ligands WS1 (red), WS4 (blue), WS7_v1 (cyan), WS7_v2 (olive), WS11 (black)


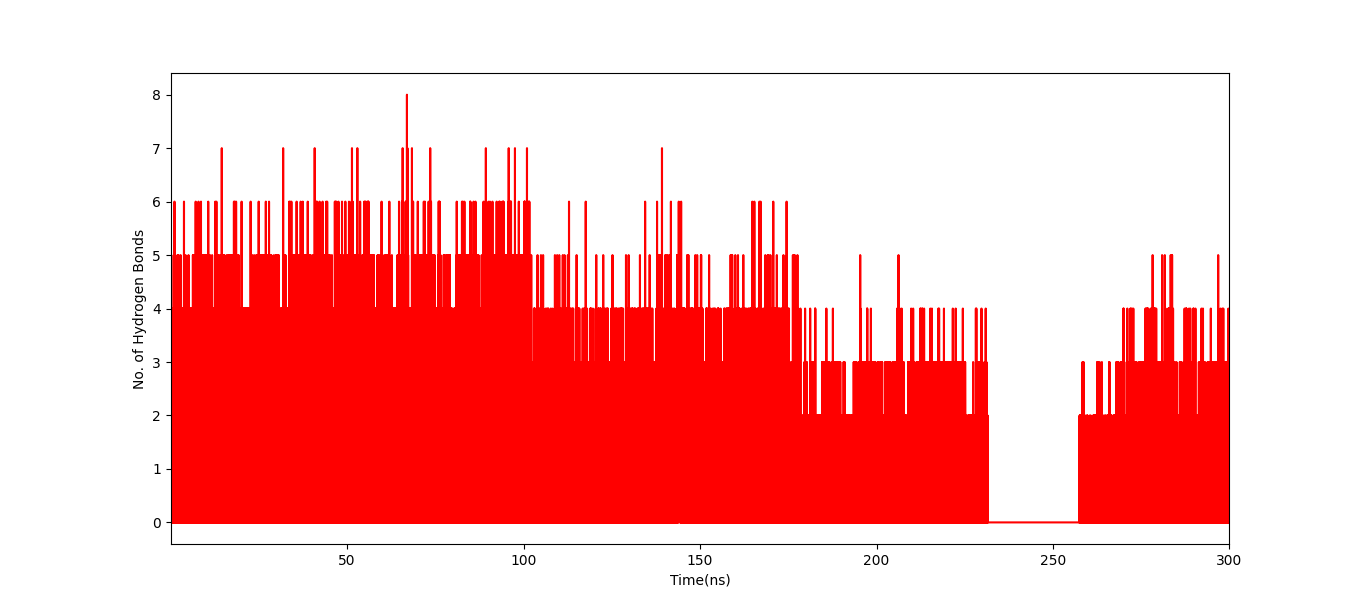


Supplementary figure 4. Number of intermolecular hydrogen bonds for the 3CL^pro^ complexes with WS1 ligand.


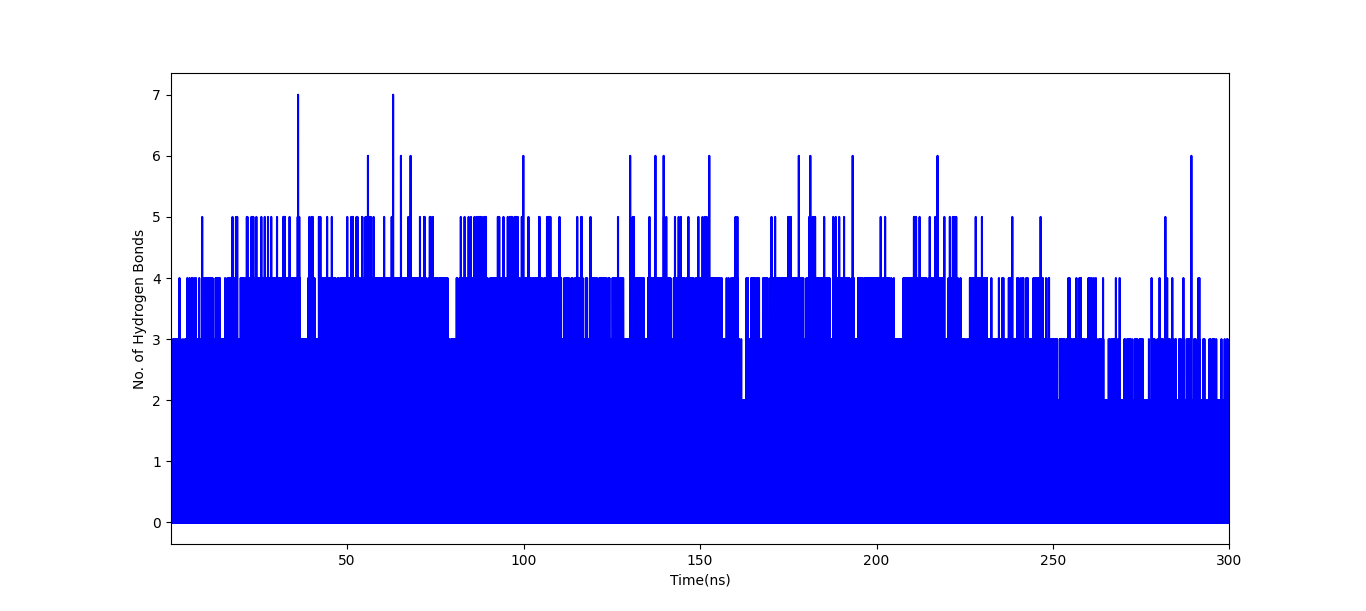


Supplementary figure 5. Number of intermolecular hydrogen bonds for the 3CL^pro^ complexes with WS4 ligand.


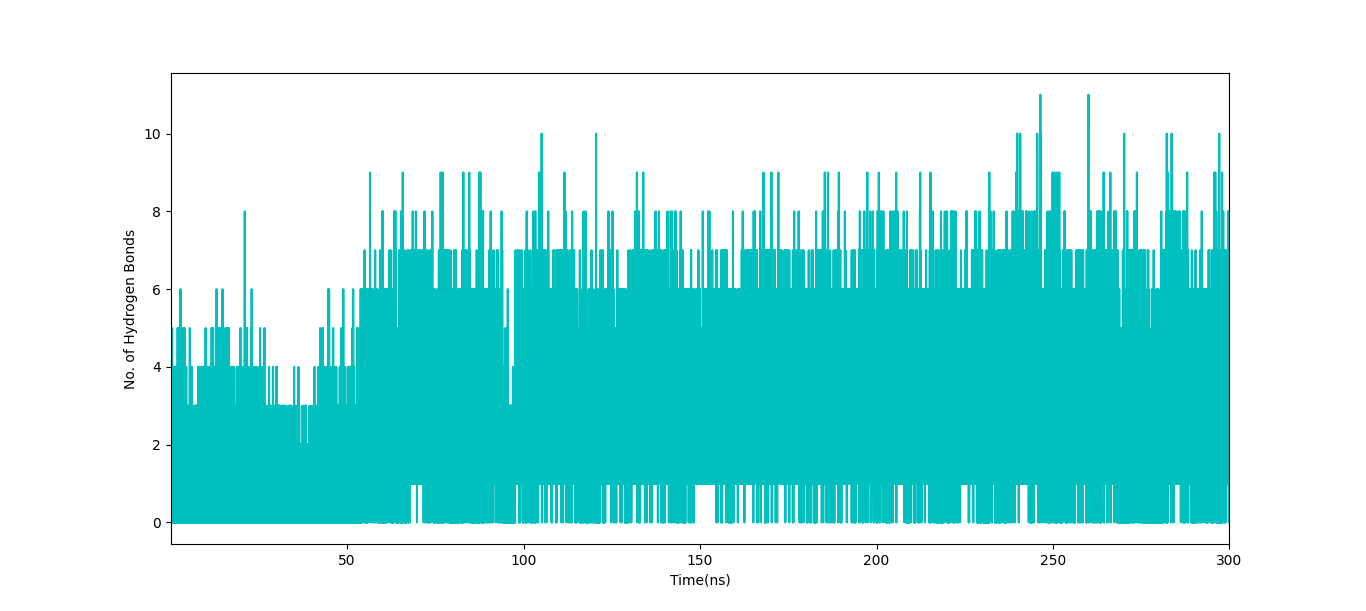


Supplementary figure 6. Number of intermolecular hydrogen bonds for the 3CL^pro^ complexes with WS7_v1 ligand.


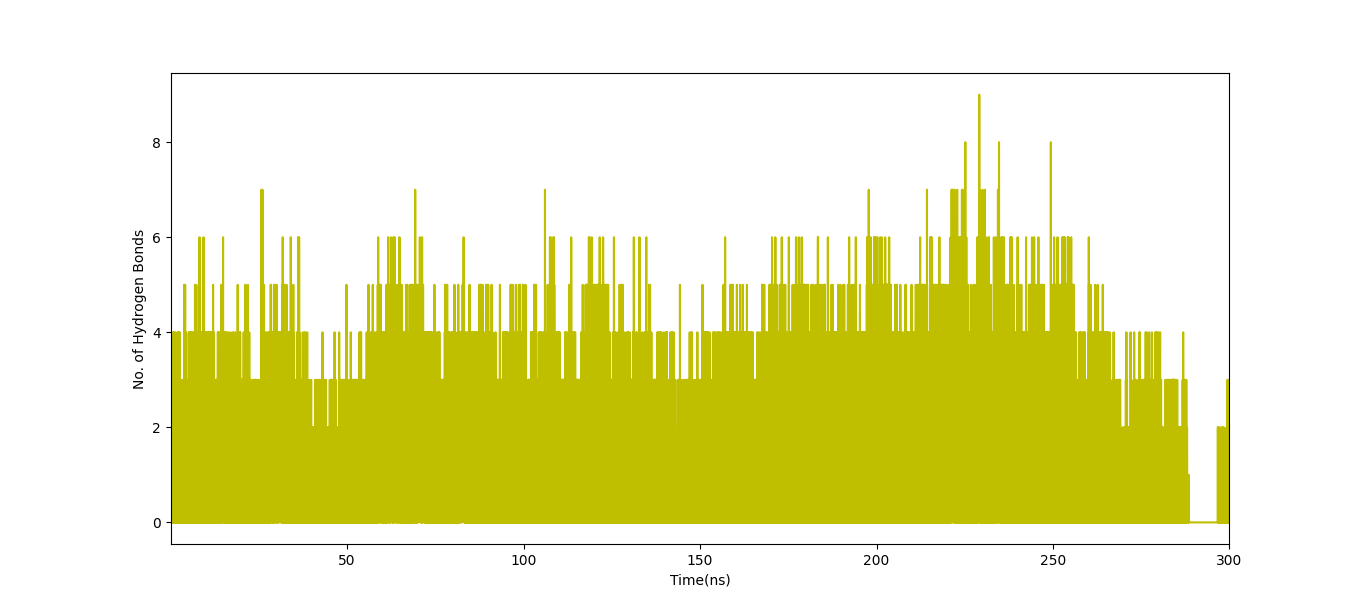


Supplementary figure 7. Number of intermolecular hydrogen bonds for the 3CL^pro^ complexes with WS7_v2 ligand.


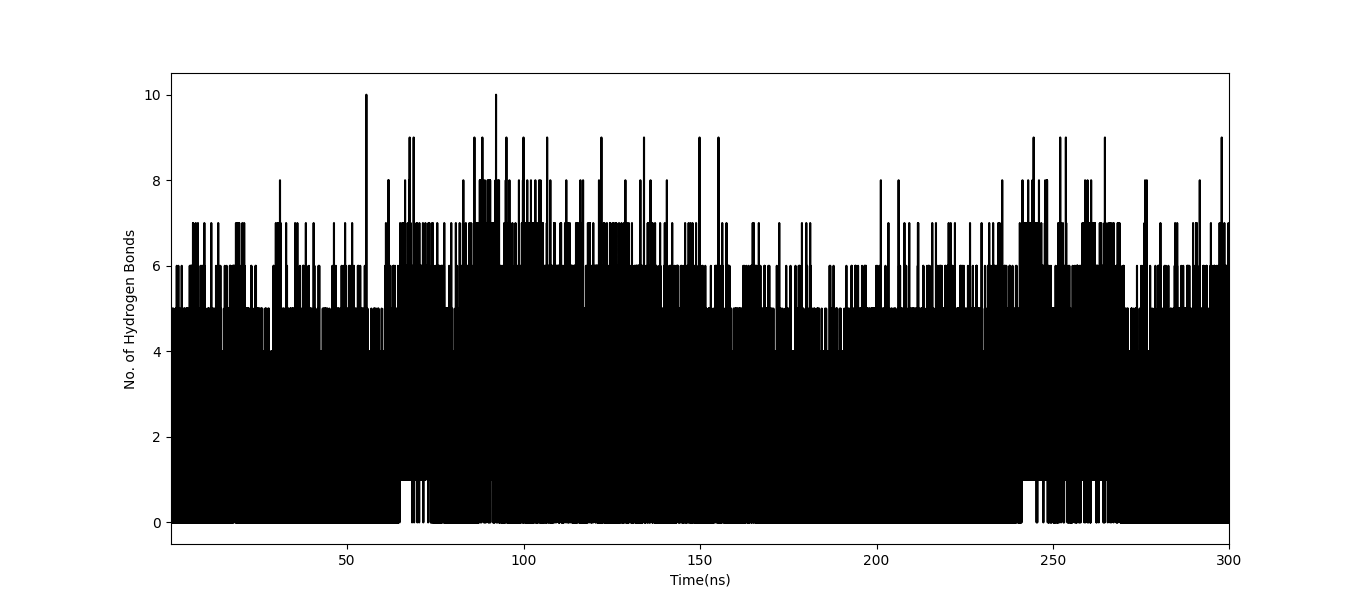


Supplementary figure 8. Number of intermolecular hydrogen bonds for the 3CL^pro^ complexes with WS11 ligand.
