# Supplementary material for: Withasomniferol C, a new potential SARS-CoV-2 main protease inhibitor from the Withania somnifera plant proposed by in silico approaches
Source: PeerJ. 2022 Jun 2;10:e13374. doi: 10.7717/peerj.13374 (PMC9167582; doi:10.7717/peerj.13374)
Supplement: Supplemental Information 8 [file peerj-10-13374-s008.docx]

| **Compound** | **Estimated binding energy (∆G_bind_)** | **MD simulation time(ns)** | **Method employed** | **Pubmed ID** |
| --- | --- | --- | --- | --- |
| Withanolide_I | -31.56 (3.74) (kcal/mol) | 100 | MM-GBSA | 33459174 |
| Withacoagulin H | −63.463(KJ/mol) | 50 | MM-GBSA | 34287986 |
| Ajugin E | −56.140 (KJ/mol) | 50 | MM-GBSA | 34287986 |
| Withacoagulin | −44.496(KJ/mol) | 50 | MM-GBSA | 34287986 |
| Withanoside II | −62.50 ± 5.25 (kcal/mol) | 100 | MM-GBSA | 32643552 |
| Withanoside IV | −81.29 ± 4.78(kcal/mol) | 100 | MM-GBSA | 32643552 |
| Withanoside V | −87.01 ± 5.01(kcal/mol) | 100 | MM-GBSA | 32643552 |
| Sitoindoside IX | −49.90 ± 4.15(kcal/mol) | 100 | MM-GBSA | 32643552 |
| Quercetin-3-rutinoside-7-glucoside | -67.767 ± 0.536(KJ/mol) | 100 | MM-GBSA | 34033891 |

Supplementary file 8: The binding free energy of the reported phytocompounds from the WS plant against the SARS-CoV-2 3CL^pro^
